# A small molecule activator of AKT does not reduce ischemic injury of the rat heart

**DOI:** 10.1186/s12967-015-0444-x

**Published:** 2015-03-01

**Authors:** Jose BN Moreira, Martin Wohlwend, Marcia NM Alves, Ulrik Wisløff, Anja Bye

**Affiliations:** K.G. Jebsen Center of Exercise in Medicine, Department of Circulation and Medical Imaging, St. Olavs Hospital, Norwegian University of Science and Technology (NTNU), Prinsesse Kristinas gt. 3, 7006 Trondheim, Norway; Norwegian Council on Cardiovascular Disease, Oslo, Norway

**Keywords:** Cardioprotection, Preconditioning, Ischemia, Apoptosis, Infarction

## Abstract

**Background:**

Activation of protein kinase AKT is required for cardioprotection by ischemic preconditioning, and transgenic overexpression of AKT protects the heart against ischemia. However, it is unknown whether acute pharmacological activation of AKT alone, using a therapeutically relevant strategy, induces cardioprotection. In this study we provide the first evidence to clarify this question.

**Methods:**

We used a recently described specific activator of AKT, the small molecule SC79, to treat rat hearts submitted to ischemia and reperfusion. Initially, isolated rat hearts were perfused with increasing doses of SC79 to verify the magnitude of AKT activation. Low and high doses were determined and used to treat hearts submitted to ischemia (35 minutes) and reperfusion (60 minutes), in a randomized and blinded design. AKT activation was verified by western immunobloting. Metabolic profile was determined by cardiac ATP content and mitochondrial enzyme activity, while cytosolic levels of cytochrome C and caspase-3 activity were used as markers of apoptosis. Ischemic injury was assessed by quantification of infarct size and cardiac release of creatine kinase and lactate dehydrogenase.

**Results:**

SC79 activated cardiac AKT within 30 minutes in a dose-dependent fashion. ATP content was largely reduced by ischemia, but was not rescued by SC79. Similarly, mitochondrial enzyme activity was not affected by SC79. SC79 administered before ischemia or at reperfusion did not prevent cytosolic accumulation of cytochrome C and overactivation of caspase-3. Finally, SC79 failed to reduce infarct size or release of cardiac injury biomarkers at reperfusion.

**Conclusion:**

We conclude that selective AKT activation by the synthetic molecule SC79 does not protect the rat heart against ischemic injury, indicating that acute pharmacological activation of AKT is not sufficient for cardioprotection.

## Background

Reestablishment of coronary blood flow after myocardial infarction (MI) is the most effective therapy to limit infarct size in humans [1]. However, cardiac injury is still observed even after successful revascularization [2], and studies showed that infarct size can be reduced by conditioning the heart with brief intermittent ischemic episodes, either before restoration of blood flow [3] or immediately at reperfusion [4].

Ischemic preconditioning, remote ischemic preconditioning and ischemic postconditioning demonstrate infarct-limiting effects in patients undergoing surgical revascularization of the myocardium [3-6], which raised a general hypothesis that intracellular mechanisms recruited by these interventions would be attractive molecular targets for pharmacological cardioprotection [7]. Based on this premise, experimental studies identified a network of proteins associated with cardioprotection by ischemic conditioning, commonly referred to as RISK (**R**eperfusion **I**njury **S**alvage **K**inases), where AKT (or Protein Kinase B, PKB) plays a central role [7]. Blocking AKT activation abrogates the benefits of ischemic preconditioning [8], remote ischemic preconditioning [9] or ischemic postconditioning [10]. Remarkably, these results have been demonstrated *in vivo* both in rodents [11] and pigs [9]. In addition, we have recently demonstrated that remote ischemic preconditioning activates AKT in the left ventricle of patients undergoing cardiac surgery [12].

Studies also showed that rodents with transgenic overactivation of AKT display reduced infarct size after coronary artery ligation when compared to controls [13-15]. These reports provided a valuable proof-of-concept, however, genetic activation of AKT in embryonic stages [15] is not a therapeutic option for humans, and preventive AKT gene therapy [13,14] is not feasible in the clinic because it is impossible to predict when a patient will suffer MI. Therefore, it is unknown whether acute activation of AKT with a therapeutically relevant strategy protects the heart against ischemia. In order to decide if cardiac AKT should be targeted in patients suffering MI, it is necessary to answer this question.

To clarify this issue, we used a novel selective activator of AKT, the small molecule SC79, recently described by Jo et al. [16]. SC79 binds specifically the PH domain (pleckstrin homology domain) of AKT, inducing a conformational change that favors its activation [16]. Importantly, their data show SC79 is cell-permeable, does not have side-effects in mice and interacts with AKT in human-derived cells, indicating that the molecule could possibly be administered intravenously in patients. Along with a detailed characterization of the molecule, the authors also reported that pretreatment with SC79 rescued neuronal cells from ischemic damage. For these reasons, we hypothesized that SC79 would protect the heart against ischemia-reperfusion injury.

To test this hypothesis, we treated isolated perfused rat hearts with low and high doses of SC79, at different time points (acutely before ischemia, immediately at reperfusion or both), and performed a comprehensive analysis of cardiac damage.

## Methods

### Animals

Female Sprague–Dawley rats (200-220 g) were used. Rats were acclimatized to our animal facilities for seven days before the experiments. Animals were housed four per cage (22°C) and had free access to standard chow and water. All experiments were approved by the Norwegian Animal Research Authority before the start of the project (FOTS#6461).

### Isolated heart perfusion

Animals were anesthetized with isoflurane, heparinized and the heart was quickly excised. The hearts were immediately perfused with the Langendorff retrograde perfusion system in constant flow (10 mL/min) with oxygenated Krebs-Henseleit buffer (37°C), comprised of 118 mM NaCl, 4.7 mM KCl, 25 mM NaHCO_3_, 5 mM glucose, 1.2 mM KH_2_PO_4_, 1.2 mM MgSO_4_ and 1.2 mM CaCl_2_ (pH 7.4). This model is extensively used in preclinical studies on myocardial protection [17] due to high reproducibility and minimal experimental dropout rate, thereby avoiding excessive use of experimental animals.

### Dose–response experiment with SC79

**Figure 1:**
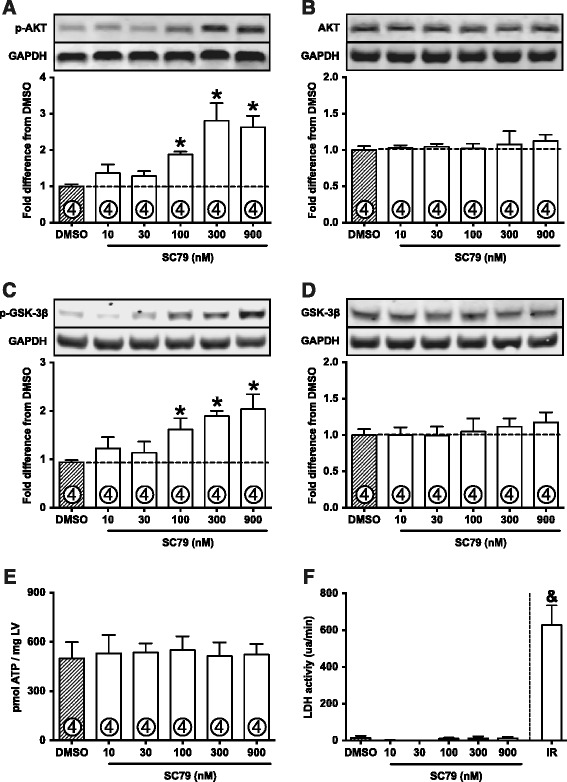
**SC79 activates cardiac AKT.** **A**: Dose–response experiment in normoxic perfused hearts treated with DMSO or SC79. SC79 activates AKT in a dose-dependent fashion, as demonstrated by phosphorylation at serine 473. **B**: Total AKT expression is not changed by SC79. **C**: Phosphorylation of GSK-3β at serine 9 (AKT-phosphorylated site). **D**: Total expression of GSK-3β. **E**: ATP levels are preserved in SC79-perfused hearts. **F**: LDH activity was not detected in SC79-treated hearts. Perfusion buffer from hearts submitted to ischemia-reperfusion (IR) were used as positive control for the LDH activity experiment. “ua/min”, units of absorbance per minute. Data are shown as mean ± standard error (SE). *, p < 0.05 vs. DMSO; &, p < 0.05 vs. all other groups.

The small molecule activator of AKT (referred to as SC79) was purchased from Tocris Biosciences (product #4635). For dose–response experiments, hearts were perfused with increasing doses of SC79 for 30 min, in a randomized fashion. The same concentration of DMSO (vehicle, 0.01%) was administered to the control group. The investigators conducting the experiments (J.B.N.M. and M.W.) were blinded to treatment assignment (DMSO or SC79). After perfusion, hearts were removed from the rig and snap-frozen in liquid nitrogen. A sample from the left ventricle was used to determine AKT activation by western immunobloting (protocol below). 100nM was adopted as low-activating dose and 300nM was adopted as high-activating dose (Figure 1A).

### SC79 treatment of ischemic hearts

**Figure 2:**
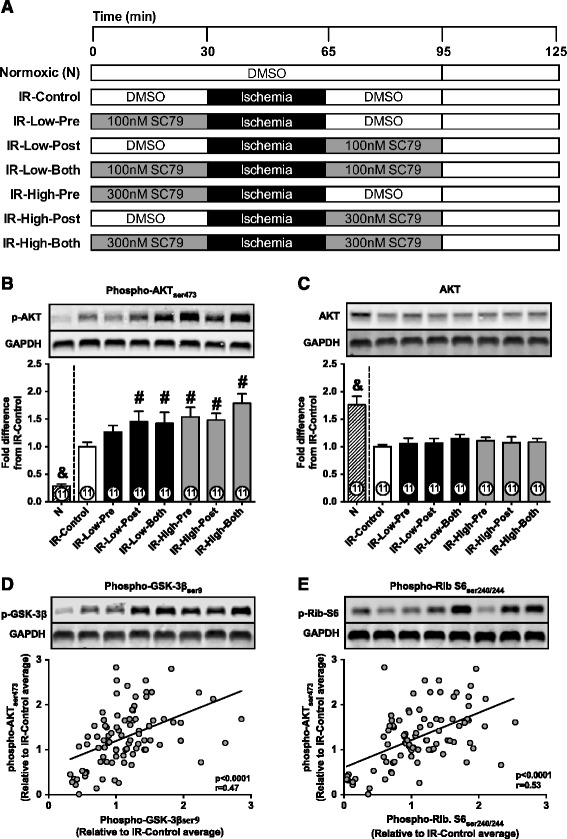
**AKT activation in ischemic hearts.** **A**: Illustrative representation of all experimental groups. “IR”, Ischemia-reperfusion. “Pre”, SC79 administered before ischemia. “Post”, SC79 administered at reperfusion. “Both”, SC79 administered before ischemia and at reperfusion. “Low” indicates 100nM SC79 and “High” indicates 300nM SC79. **B**: AKT phosphorylation in normoxic-perfused (N) or ischemic hearts (IR) treated with DMSO or SC79. **C**: Total AKT expression; **D**: Correlation between AKT phosphorylation and GSK-3β phosphorylation. **E**: Correlation between AKT phosphorylation and Ribosomal-S6 phosphorylation. The Pearson correlation test was used to analyze the correlations shown in panels **D** and **E**. Number of animals analyzed per group is displayed within the bars. Western blot bands displayed in panels **D** and E are disposed in the same group order as in panels **B** and **C**. Data are shown as mean ± SE. #, p < 0.05 vs. IR-Control; &, p < 0.05 vs. all other groups.

Low- and high-activating doses of SC79 were used to treat hearts submitted to ischemia and reperfusion (IR). Treatments were performed either before ischemia, at reperfusion or both. Same concentration of DMSO (0.01%) was added to the buffer of normoxic perfused hearts and ischemic control hearts. Experimental groups are illustrated in Figure 2A and described as: (group “N”) normoxic hearts, perfused continuously for 125 min; (IR-Control) ischemic DMSO-treated control group, hearts perfused for 30 min with normoxic buffer, followed by 35 min of no-flow ischemia (37°C) and 60 min reperfusion; (IR-Low-Pre) hearts received low-dose SC79 for 30 min before ischemia; (IR-Low-Post) hearts received low-dose SC79 for the initial 30 min of reperfusion; (IR-Low-Both) hearts received low-dose SC79 before ischemia and at reperfusion; (IR-High-Pre) hearts received high-dose SC79 for 30 min before ischemia; (IR-High-Post) hearts received high-dose SC79 for the initial 30 min of reperfusion; (IR-High-Both) hearts received high-dose SC79 before ischemia and at reperfusion. Each group consisted of 11 rats (see “Statistical analysis” for details on power calculation). Experiments were conducted in a randomized and blinded fashion. DMSO and SC79 were aliquoted before the start of the study, when the aliquots received unique five-digit codes and were kept frozen until the experiment. Each code was randomly assigned to one rat. The identity of the vials was controlled by two colleagues not involved in the study, and was revealed to the investigators after data were collected from all animals. No animal was arbitrarily excluded from the study.

### Cardiac enzyme release and infarct size

The buffer perfusate was collected immediately before ischemia and at reperfusion for quantification of cardiac enzyme release. Creatine kinase (CK) was quantified by ELISA (Abcam, kit #ab187396) and lactate dehydrogenase (LDH) activity was measured with a colorimetric assay (Thermo Scientific, kit #88953) according to manufacturer’s protocol. The heart was removed from the rig at the end of reperfusion (60 min), sliced into 2 mm-thick sections and stained with 2% triphenyltetrazolium chloride (TTC, diluted in phosphate buffer). Stained slices were scanned between glass slides on a black background. Infarct area was quantified with the ImageJ software and is presented as percentage of total area of the left ventricle.

### Western immunobloting

Left ventricular samples frozen in liquid nitrogen at the end of reperfusion were used for these analyses. Samples were homogenized in RIPA buffer containing protease and phosphatase inhibitors (Sigma-Aldrich), and centrifuged (20 min, 4°C, 16000 g) to remove pelleted debris. Total protein content was measured and sample loading buffer was added, followed by heating (10 min). Proteins were separated by gel electrophoresis (Bolt precast gels, Life Technologies) and transferred to nitrocellulose membranes. An internal control sample was added to each gel in order to allow comparison among samples in different membranes. Membranes were blocked with 5% bovine serum albumin for 1 h, incubated with primary antibodies overnight (4°C) and with secondary antibodies (LICOR Biosciences, UK) for one hour. The following primary antibodies were used (numbers indicate Cell Signaling product code): AKT (#4685), phospho-AKT_ser473_ (#4060), GSK-3β (#12456), phospho-GSK-3β_ser9_ (#5558), Ribosomal-S6 protein (#2317) and phospho-Ribosomal-S6_ser240/244_ (#5364). GAPDH (Glyceraldehyde 3-phosphate dehydrogenase) was adopted as loading control (Pierce Antibodies, #MA5-15738). The representative bands shown in the figures were not modified or rearranged, and illustrate one original gel used in the analysis. Samples remaining from these experiments were frozen in liquid nitrogen and stored at −80°C.

### ATP content and activity of mitochondrial enzymes

For ATP determination, samples were homogenized and deproteinized with 5% trichloroacetic acid, the pH was corrected to 7.4 with Tris-acetate buffer and samples were immediately analyzed with a bioluminescence assay using recombinant firefly luciferase (Life Technologies, kit #A2206). Activity of citrate synthase and 3-hydroxyacyl-CoA dehydrogenase (HADH) were analyzed with colorimetric assays, as previously described [18].

### Cytosolic levels of cytochrome C

Cytosolic and mitochondrial fractions were isolated by differential centrifugation (Thermo Scientific, kit #89801). Both fractions were analyzed by western immunobloting using an antibody against Cytochrome C (Cell Signaling, #11940). VDAC (Voltage-dependent anion channel, Abcam, #ab14734) was used as mitochondrial loading control and GAPDH was used as cytosolic loading control.

### Caspase-3 activity

Caspase-3 activity in left ventricle homogenates was measured using a specific substrate (DEVD, amino acid sequence Asp-Glu-Val-Asp) conjugated with 7-amino-4-methylcoumarin probe, which emits fluorescence (excitation/emission, 342/441 nm) after proteolytic cleavage (Life Technologies, #E-13183). A caspase-3 inhibitor (Ac-DEVD-CHO) was used in a separate well to confirm that activity corresponded specifically to caspase-3.

### Statistical analysis

The entire strategy for statistical analysis was defined before the start of the study. In order to calculate sample size, we conducted a pilot experiment (n = 6) and verified mean infarct size (27.1% as measured in TTC-stained slices) and variation (standard deviation = 7.6%) in the exact conditions of the study. We then hypothesized a reduction of 30% in infarct in size (from 27.1 to 18.9%), which is a value considered to be clinically significant in human clinical trials [19]. Using these data, the power analysis (SPSS SamplePower, IBM) indicated that 11 animals per group would be necessary to detect a significant effect with 80% power and α < 0.05. Normal distribution of the data was confirmed by the Shapiro-Wilk test. The Grubbs’ test was used to detect outliers (α < 0.05). Groups were compared by one-way analysis of variance (ANOVA) followed by the Fisher post hoc test. A p-value below 0.05 was considered significant.

## Results

### Cardiac AKT activation by SC79

Normoxic perfused hearts receiving SC79 for 30 min displayed a dose–response pattern of AKT activation (Figure 1A), as assessed by phosphorylation at serine 473. Activation was not significant with 10 or 30nM doses. 100nM SC79 increased AKT phosphorylation by 1.88 fold above DMSO group (p = 0.028), while maximum effect was observed with a dose of 300nM (2.81 fold increase in AKT phosphorylation, p = 0.0001) (Figure 1A). 900nM SC79 did not increase AKT phosphorylation above that observed with 300nM. Total expression of AKT was not altered by SC79 (Figure 1B). In order to confirm AKT overactivation, we assessed phosphorylation of GSK-3β at serine 9, a direct target of AKT. Indeed, activation of AKT by SC79 led to phosphorylation of GSK-3β (Figure 1C), without changes in total GSK-3β abundance (Figure 1D).

We also asked the question of whether SC79 itself could be toxic to the heart. To clarify this point we assessed cardiac ATP levels and LDH release from the hearts perfused with SC79 (10-900nM) under normoxic conditions. Importantly, the doses we used of SC79 were not harmful to perfused hearts, since ATP levels remained preserved (Figure 1E) and LDH activity was not detected in the perfusion buffer (Figure 1F), indicating that no damage was caused by the compound.

The results described above provided the doses to be used in the protocols with ischemic hearts. The protocols are illustrated in Figure 2A. Phosphorylation of AKT was increased (Figure 2B) in ischemic hearts when compared to normoxic perfused heart, while total AKT abundance was reduced (Figure 2C), as previously demonstrated [20]. The increase in phosphorylation was observed even when phospho-AKT levels were not corrected for total AKT. Overactivation of AKT by SC79 was maintained until the end of reperfusion, except in the group receiving a low dose of the compound exclusively before ischemia (Figure 2B, “IR-Low-Pre” group). Phosphorylation of GSK-3β (direct target of AKT) and Ribosomal-S6 protein (indirect downstream target of AKT) correlated significantly with AKT phosphorylation in our cohort (Figures 2D and E). Total expression of GSK-3β and Ribosomal-S6 protein were not altered by ischemia or SC79 (not shown).

### ATP content and mitochondrial enzyme activity

**Figure 3:**
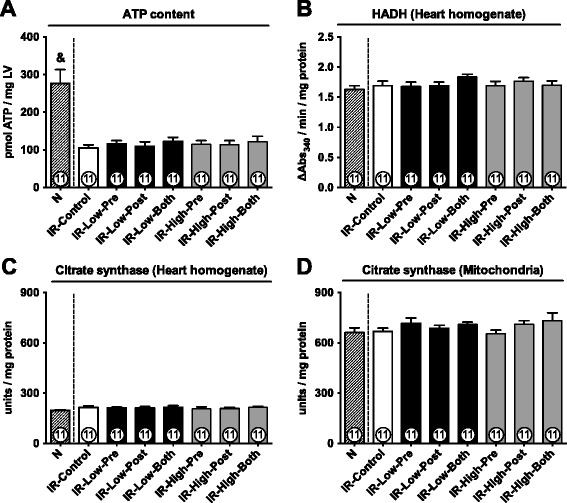
**Cardiac ATP content and mitochondrial enzyme activity.** **A**: Cardiac ATP content at the end of reperfusion. LV, left ventricle. **B**: 3-hydroxyacyl-CoA dehydrogenase (HADH) activity. **C**: Citrate synthase activity in heart homogenates. **D**: Citrate synthase activity in isolated mitochondria. Data are shown as mean ± SE. &, p < 0.05 vs. all other groups.

Disrupted energy metabolism is a hallmark of ischemia and an important endpoint in studies on cardioprotection [4,21]. ATP content was largely reduced in hearts submitted to ischemia (Figure 3A). ATP depletion was similar among IR-Control and all SC79-treated groups (Figure 3A). We also assessed maximal activity of mitochondrial enzymes HADH (essential for beta-oxidation of fatty acids) and citrate synthase (pace-making enzyme for the first step of the Krebs cycle). Surprisingly, activity of HADH (Figure 3B) and citrate synthase (Figure 3C and D) were not affected by ischemia, suggesting that the reduced ATP content was due to dysfunction of mitochondrial complexes rather than loss of mitochondria. SC79 did not affect activities of HADH (Figure 3B) or citrate synthase (Figure 3C and D).

### Apoptosis

**Figure 4:**
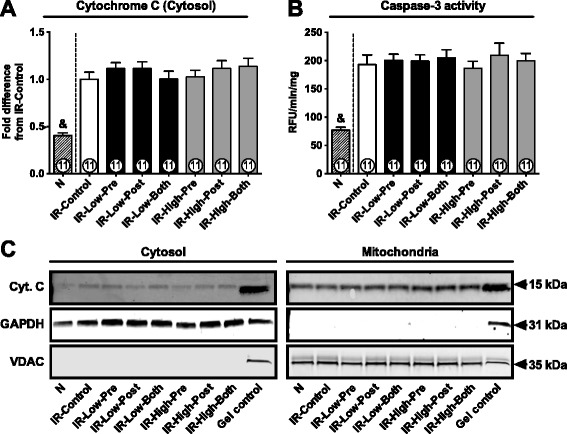
**Cytosolic cytochrome C and caspase-3 activity.** **A**: Cytochrome C abundance in cytosol. **B**: Proteolytic activity of caspase-3. RFU/min/mg, relative fluorescence unit/minute/mg protein. **C**: Original representative western blots of cytochrome C (Cyt. C). GAPDH and VDAC were used as cytosolic and mitochondrial loading controls, respectively. A heart homogenate sample was used as gel internal control. Data are shown as mean ± SE. &, p < 0.05 vs. all other groups.

Release of cytochrome C from mitochondria to cytosol activates caspase-3, which is a central mechanism in the execution of cardiomyocyte cell death by apoptosis in humans [19,22]. Our data show that activation of AKT by SC79 did not prevent accumulation of cytosolic cytochrome C (Figure 4A) or overactivation of caspase-3 (Figure 4B) caused by ischemia. Original representative western blots of cytochrome C are shown in Figure 4C.

### Ischemic damage and infarct size

**Figure 5:**
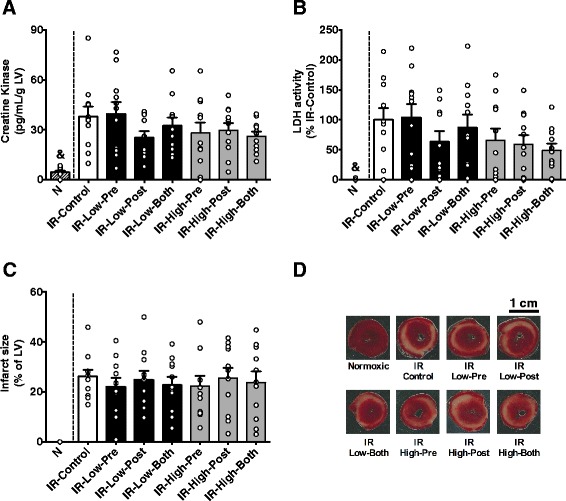
**Ischemic injury.** **A**: Abundance of creatine kinase in the perfusion buffer at reperfusion. **B**: Activity of lactate dehydrogenase (LDH) in the perfusion buffer at reperfusion. **C**: Infarct size as measured by histology after staining with Triphenyltetrazolium chloride. **D**: Representative histological sections of the left ventricle; Each dot within the bars represents one animal of the group. Note that IR-Low-Post shows 10 animals in panels “**A**” and “**B**” because one rat was detected as an outlier for these measurements. Other groups show data from all 11 animals. Data are shown as mean ± SE. &, p < 0.05 all other groups.

Cardiac release of intracellular enzymes and infarct size are important predictors of prognosis and mortality after MI, and are used as primary outcome in clinical studies [5,6]. Abundance of CK and LDH was below detection limit in most of the hearts submitted to normoxic perfusion (Figures 5A and B). SC79 administered before ischemia or at reperfusion did not significantly reduce cardiac release of CK (Figure 5A) and LDH (Figure 5B). Similarly, SC79 did not promote survival of myocardial tissue after ischemia, as evidenced by the data on infarct size (Figure 5C). Figure 5D shows representative cross sections of the left ventricle after TTC staining.

## Discussion

This is the first study to determine whether acute pharmacological activation of AKT protects the myocardium against ischemic damage. Here we show that SC79, a small molecule activator of AKT, fails to reduce ischemic injury of the heart. SC79 administered in low or high doses, before or after ischemia, neither preserved cardiac ATP content nor prevented overactivation of caspase-3, a central player in apoptosis. Consequently, we did not observe significant reduction of infarct size or cardiac injury biomarkers (CK and LDH). Our data are surprising and differ from previous studies with genetic models of cardiac AKT overexpression [13-15]. Fundamental differences between the models (transgenic vs. small molecule) certainly account for the distinctive findings, and our results demonstrate that the protection observed in transgenic animals is not reproduced by a potentially therapeutic molecule (SC79). Two crucial issues to be considered are the strategy for AKT activation and timing. In our study, overactivation of AKT was induced acutely by a small molecule, via a post-translational mechanism where AKT becomes more susceptible to phosphorylation without changes in total expression [16] (Figures 1 and 2). Differently, Matsui et al. studied 9 to 12-week old transgenic mice with cardiac-specific expression of an active mutant form of AKT (myr-AKT) [15], while in two other studies cardiac overexpression of myr-AKT was induced by transfection with recombinant adenovirus at least 48 h before induction of ischemia [13,14]. The same authors reported that the myr-AKT transgene caused remarkable changes in cardiac gene expression profile [23], inducing differential expression of several genes previously unrelated to AKT signaling and not involved in canonical signaling downstream of AKT. This transcriptional response is thought to mediate the cardioprotective mechanism in these mice because phosphorylation of canonical AKT targets (e.g. BAD and GSK-3β) was not affected in the model [23], or in animals transfected with myr-AKT in adulthood [14]. On the other hand, hyperactivation of AKT by SC79 in the short timescale of our study (30-60 min) is unlikely to exert such profound changes on gene transcription, which might account for the lack of cardioprotection. Additionally, overexpression of myr-AKT over a 48 h period improves mitochondrial efficiency and ATP production in isolated cardiomyocytes under normoxic conditions [24], which contributed to increased tolerance to hypoxia at a later time. However, our results show that short-term activation of AKT with SC79 did not increase ATP levels at reperfusion or improve mitochondrial enzyme activity in ischemic hearts (Figure 3). Moreover, cardiac-specific overexpression of active AKT promotes angiogenesis and increases cardiac vascularization in adult animals [25]. This is clearly a chronic adaptation that confers a huge advantage in the context of ischemia-reperfusion injury. It is also important to notice that although these studies provided a valuable and robust proof-of-concept, their clinical implications are limited. Genetic activation of AKT in these studies was achieved either during embryonic stages or induced as a preventive measure, days or weeks before the infarction. Differently, the clinical scenario of acute MI is totally unpredictable and therefore requires intervention immediately before ischemia (i.e. at the onset of symptoms) or most preferably at reperfusion (i.e. during percutaneous coronary intervention or coronary bypass graft surgery). This requirement is met by cell-permeable small molecules with rapid action, which justifies the design of our protocols (Figure 2A).

The rationale presented up to now is compelling to discern our findings from those obtained with genetic manipulation of AKT, but it is still insufficient to explain why treatment with SC79 before ischemia or at reperfusion failed to mimic the effects of ischemic pre- and postconditioning, respectively. Ischemic conditioning is an acute cardioprotective intervention that induces post-translational activation of AKT, which is mirrored by SC79 both in magnitude and timing [26] (Figures 1A and 2B). In this sense, it was shown that mice lacking AKT cannot be protected by ischemic preconditioning [11], what is corroborated by the findings that pharmacological blockage of Pi3k-mediated AKT activation impedes the effects of ischemic conditioning [8-10]. Despite these facts, other studies also proved that cardioprotection is abolished when inhibitors of ERK1/2 (Extracellular signal-regulated kinases) [27], JAK (Janus kinase) [28] or protein kinase C [29] are applied together with ischemic conditioning. The same results were observed after blockage of adenosine receptors [30], mitochondrial potassium channels [31], protein kinase A [32] or other less studied proteins. These studies form a strong body of evidence showing that ischemic conditioning induces a highly orchestrated rearrangement of intracellular signaling, where activation of many individual players seems to be necessary. Although AKT clearly is one of those players, it does not seem to be sufficient. In this regard, it is also important to notice that most studies investigated the role of Pi3k-AKT signaling in cardioprotection by blocking the entire cascade downstream of Pi3k, using the non-selective inhibitors Wortmannin or LY294002 [8,9,10,13]. These chemicals are known to block cellular events independent of AKT [33], and a study suggested a Pi3k-mediated, AKT-independent mechanism is also implicated in cardioprotection against ischemia [34]. It has also been suggested that chronic (life-long), excessive activation of AKT could lead to negative feedback inhibition of Pi3k and indirectly block protective signaling in the heart [34]. This is very unlikely to be the case in our study because the stimulus for AKT activation was acute, and the magnitude of activation reported here is largely similar to what is obtained after ischemic preconditioning and remote ischemic preconditioning, as mentioned above.

One could also ask whether unspecific molecular signaling induced by SC79 could have blocked a potential cardioprotective effect of the drug. The only evidence regarding specificity of SC79 is provided by the original study that described the molecule, and the findings show that SC79 binds specifically the PH domain of Akt [16]. They also show the effect of SC79 does not require insulin, activation of signaling upstream of Akt (Pi3k) or membrane receptors (e.g. G-protein coupled receptors). These facts indicate that action of SC79 is totally independent of blood serum factors. Additionally, four other studies have applied SC79 in vitro or in vivo, and have not reported evidence that the molecule has off-target effects [35-38]. However, considering that these studies were not designed with the clear goal to address specificity of SC79, we cannot completely rule out the possibility that SC79 interacts with proteins other than AKT and interferes with cardioprotective signaling indirectly. Nevertheless, a recent study showed that SC79 alone induced glucose uptake in lymphocytes by promoting translocation of GLUT1 (Glucose Transporter 1) to the cell membrane [38]. In the context of cardiac ischemia, stimulation of glucose uptake without compensatory increase of intracellular glucose oxidation could be detrimental and prevent a potential benefit of SC79 [39,40]. On the other hand, cardioprotection is detectable when higher glucose uptake is matched by increased glucose oxidation and consequent production of ATP, which is the scenario observed in ischemic preconditioning [41], exercise training [42,43] or after chemical activation of AMPK (AMP-activated protein kinase α) [44], a well-known stimulator of glucose uptake and oxidation.

In summary, our discovery shows that acute administration of SC79 does not recapitulate the benefits of chronic AKT activation shown in previous studies, and also suggest that the pursuit of pharmacological cardioprotection may require concomitant activation of multiple intracellular targets, which is an endeavor for future studies in the field.

Finally, it is important to emphasize that our findings by no means challenge the mechanistic hypotheses of the studies mentioned in this discussion. The hypothesis of the present study has never been tested before, particularly because SC79 is the first specific pharmacological activator of AKT described in the literature [16]. Using this therapeutically relevant strategy, we sought to investigate outcomes that are used in the clinic (release of cardiac injury biomarkers and infarct size). However, a protective effect of SC79 was not confirmed.

## Conclusion

We conclude that acute pharmacological activation of AKT alone by SC79 is not a sufficient condition to evoke cardioprotection against ischemia in rats.

### Limitations

All rats used in this study were young and without comorbities, which is a condition that is the relatively different from that of most infarcted patients. These patients are usually older and have at least one major comorbidity, such as diabetes or hypertension. However, age and comorbidities are known to interfere with AKT activation and cardioprotection [26,45], and would therefore be confounding factors in our analysis.

## Abbreviations

AKT: Protein Kinase B
ATP: Adenosine triphosphate
MI: Myocardial infarction
RISK: Reperfusion injury salvage kinases
PH domain: Pleckstrin homology domain
DMSO: Dimethyl sulfoxide
ELISA: Enzyme-linked immunosorbent assay
CK: Creatine kinase
LDH: Lactate dehydrogenase
TTC: Triphenyltetrazolium chloride
HADH: 3-hydroxyacyl-CoA dehydrogenase
GAPDH: Glyceraldehyde 3-phosphate dehydrogenase
VDAC: Voltage-dependent anion channel
ANOVA: analysis of variance
BAD: Bcl-2-associated death promoter
ERK1/2: Extracellular signal-regulated kinases
JAK: Janus kinase. LV, left ventricle
